# Necrotizing Enterocolitis and Neurodevelopmental Impairments: Microbiome, Gut, and Brain Entanglements

**DOI:** 10.3390/biom14101254

**Published:** 2024-10-04

**Authors:** Cuilee Sha, Zhaosheng Jin, Stella Y. Ku, Ann S. Kogosov, Sun Yu, Sergio D. Bergese, Helen Hsieh

**Affiliations:** 1 Department of Pharmacological Sciences, Stony Brook University, 100 Nicolls Road, Stony Brook, NY 11794, USA; cuilee.sha@stonybrookmedicine.edu; 2 Center for Nervous System Disorders, Stony Brook University, 100 Nicolls Road, Stony Brook, NY 11794, USA; 3 Department of Anesthesiology, Stony Brook Medicine, 101 Nicolls Road, Stony Brook, NY 11794, USA; zhaosheng.jin@stonybrookmedicine.edu; 4 Stony Brook University, 100 Nicolls Road, Stony Brook, NY 11794, USA; 5 Renaissance School of Medicine, Stony Brook University, 100 Nicolls Road, Stony Brook, NY 11794, USA; 6 Department of Surgery, Stony Brook Medicine, 101 Nicolls Road, Stony Brook, NY 11794, USA

**Keywords:** gut–microbiota–brain, microbiome, nutrition, development, necrotizing enterocolitis (NEC), inflammatory bowel disease, neurological disorders

## Abstract

There is significant communication and interdependence among the gut, the microbiome, and the brain during development. Diseases, such as necrotizing enterocolitis (NEC), highlight how injury to the immature gastrointestinal tract leads to long-term neurological consequences, due to vulnerabilities of the brain in the early stages of life. A better understanding of the developing gut–microbiota–brain axis is needed to both prevent and treat the devastating consequences of these disease processes. The gut–microbiota–brain axis is a bidirectional communication pathway that includes metabolic, nervous, endocrine, and immune components. In this review, we discuss gut development, microbiome colonization and maturation, and the interactions that influence neurodevelopment in the context of NEC. We describe the components of the gut–brain axis and how the microbiome is an integral member of this relationship. Finally, we explore how derangements within the microbiome and gut–microbiota–brain axis affect the normal development and function of the other systems and long-term neurodevelopmental consequences for patients.

## 1. Introduction

All throughout life, the gastrointestinal (GI) system and central nervous system (CNS) maintain an important bidirectional connection, referred to as the gut–brain axis. While the brain controls all peripheral organs through the autonomic nervous system, the ways in which the gut influences the brain are becoming more apparent and critical to study scientifically. The influence of one system on the other is especially highlighted during disease, where nausea and vomiting or abnormal bowel changes accompany neurological disorders and, conversely, where depression or anxiety are found in patients with inflammatory bowel disease (IBD) [1,2,3]. In the pediatric population, early insults to the GI tract (i.e., necrotizing enterocolitis, bacterial dysbiosis, or imbalance) can lead to long-lasting neurodevelopmental delay and autism-like symptoms [4,5,6]. The relationship between these two systems needs to be better understood in early life, when the gut and nervous system develop in parallel and are particularly vulnerable to injury due to their immaturity [7,8].

The gut microbiome, which includes the microorganisms that comprise the microbiota and their environment, plays an important role in the gut–brain axis, especially during development. Microbial-derived intermediates serve as neurodevelopmental and neuroimmune signals to the immature central nervous system [9]. These include microbial-derived short chain fatty acids (SCFAs), which can promote the maturation of microglia [10], resident innate immune cells of the brain that are instrumental to the refinement of cortical neuronal circuits and the development of other glial cells in the brain [11]. Secondary bile acids from bacterial fermentation help promote differentiation of regulatory T (Treg) cells in the brain that modulate the adaptive immune response. Other substances, such as tryptophan metabolites, have also been described as modulating inflammatory process in the CNS [12]. Conversely, signals from the brain can affect the microbiome by altering gut motility, permeability, and function. For example, the autonomic nervous system, which is modulated centrally by the pituitary and hypothalamus, can regulate and alter bowel transit time, goblet cell function, and mucosal permeability of the gut [13].

A healthy gut–microbiota–brain axis is essential for normal neurodevelopment in early life, and disruption of the microbiome may adversely affect neurocognitive development. Preterm infants, for example, are at higher risk of gastrointestinal dysbiosis, which is defined by an imbalance of microorganisms in the microbiome, as well as colonization by pathogenic microorganisms because of antibiotic use and prolonged hospital course. This dysbiosis may compromise the gut epithelial barrier and can result in necrotizing enterocolitis (NEC), a devastating inflammatory disease of the intestines that results in widespread inflammation [14,15]. The resulting systemic inflammatory processes can adversely affect normal neurodevelopment in immature brains. Interestingly, microbiome-derived SCFAs also support intestinal epithelial cell tight junctions and reduce gut permeability [16], which may help protect against NEC. To better isolate potential therapeutic targets for NEC and various pediatric diseases that affect the gut–microbiota–brain axis, it is important to understand this axis, the signals that exist along it, and how everything interacts during development.

Therefore, the aim of this review is to describe the gut–microbiota–brain axis in early development. We will explore gut development, microbiota colonization and maturation, the gut–brain and gut–microbiota–brain axes, and current literature regarding the impacts of gut–microbiota–brain axis dysfunction on neurodevelopment in the context of prematurity and NEC.

## 2. Gut Microbiome Development

The development of the GI tract is a complex process that relies on successful interactions with various organ systems, notably the nervous and immune systems. Embryologic interactions between the GI and nervous systems are essential for proper development, and any disruptions may have significant implications for their function later in life. A critical aspect of GI tract development is the formation of tight junctions, which link adjacent epithelial cells and regulate permeability, maintaining the integrity of the intestinal barrier [17]. These tight junctions are not fully developed in preterm infants and can be affected by microbiome composition. Furthermore, the mucus layer, which normally prevents intestinal bacterial translocation, is decreased in premature infants [18]. Underdevelopment of these innate protective mechanisms in premature infants leaves the GI tract highly susceptible to invading pathogens.

### 2.1. Colonization

Although recent findings suggest that colonization of the GI tract may occur before birth [19], perinatal and neonatal exposures to microbes are critical. These neonatal exposures include microbiota acquired from the mother during delivery and the external environment, including diet and antibiotic use [20,21]. This colonization differs further between infants born via vaginal and cesarean deliveries. Kim et al. found increased stool microbiota diversity in vaginally delivered neonates, which was not observed in neonates delivered by cesarean section [22]. The microbiome functional profiles of these vaginally delivered neonates demonstrated higher expression of carbohydrate, nucleotide, and amino acid metabolism genes, whereas the profiles from the cesarean neonates demonstrated higher expression of lipopolysaccharide (LPS) biosynthesis genes. In some studies, cesarean-delivered neonates may have nosocomial organism colonization [23], such as *Clostridium difficile* [24]. While some reports suggest that the difference in gut microbiota normalizes by 6 weeks after delivery [25], others have identified differences that persist for several years [26]. Subtle differences in colonization may have long-term implications for microbiota health and the gut–brain axis.

### 2.2. Microbiome Development and Maturation

The gut microbiota matures in parallel with the rest of the body. Microbiome profile variability between individual infants stabilizes during the first 3 years of life [27,28]. Bacteroidetes and other gram-negative organisms predominate in early pediatric microbiomes (Table 1), while Firmicutes prevail in adults [28]. Using functional metagenomics, pediatric microbiomes demonstrate increased expression of metabolic pathways for vitamin (folate, riboflavin, pyridoxine) biosynthesis and glycan/amino acid (valine, leucine, isoleucine) degradation. In contrast, adult microbiomes demonstrate increased carbohydrate metabolism and amino acid biosynthesis [27,28].

***Preterm*** birth results in numerous physiological and clinical disruptions, including altered exposure to maternal microbiome, GI track, and immune system immaturity, as well as subsequent nosocomial exposures [30]. It has been observed that the microbiome in preterm infants has a higher abundance of organisms including *Staphylococcaceae*, *Streptococcaceae,* and *Enterobacter* [30,31], while being deficient in *Bifidobacterium* and *Lactobacilli* (Table 2) [30,32]. Additionally, higher Gammaproteobacterium levels have also been observed in association with antenatal steroid administration [30]. As preterm infants age, the relative abundance of bacilli decreases, while the abundances of *Clostridium* and Actinobacteria (including *Bifidobacterium*) rise [30,33].

### 2.3. Nutritional Microbiome Modulators

Maternal and perinatal ***antibiotic*** administration can also modulate microbiome development in neonates. Tapiainen et al. prospectively evaluated the stool microbiome of neonates, who were stratified according to perinatal antibiotic exposure (no exposure, maternal exposure only, neonatal exposure only, or both maternal and neonatal exposure) [34]. Antibiotic-naive newborns had significantly increased Bacteroides and decreased Firmicutes levels 3 days after delivery, which persisted at 6 months of age (Table 2). Li et al. found that, in infants whose mothers did not receive perinatal antibiotics, the neonatal stool microbiome closely resembled the maternal microbiome, whereas neonates with perinatal antibiotic exposure had microbiomes that differed considerably from the maternal microbiome [35].

**Figure 1:**
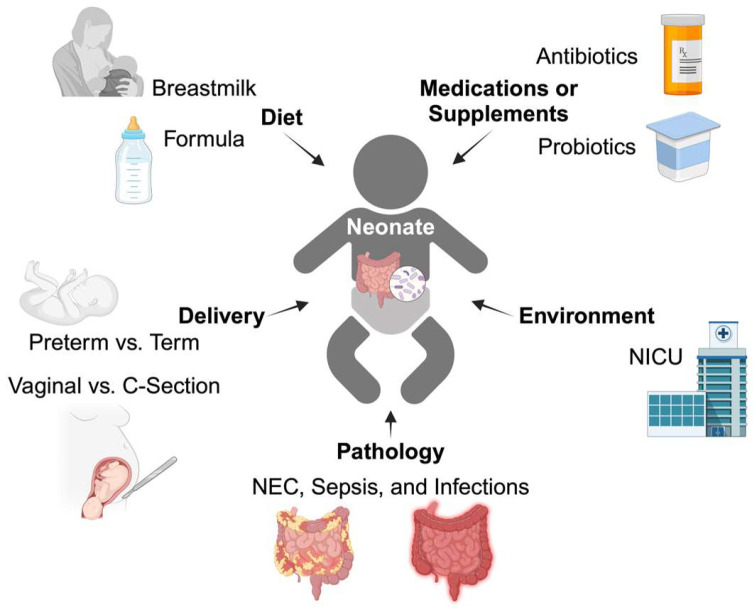
Early neonatal exposures that affect the gut microbiome. The neonatal microbiome, as it undergoes colonization, is particularly vulnerable to environmental exposures. Notable exposures include delivery conditions, diet, medications or supplements, environment, and pathology. Specifically, delivery conditions include the neonate’s gestational age (preterm <37 weeks vs. term) and mode of delivery (vaginal vs. C-section). A neonatal diet is largely either breastmilk or formula, the latter of which can include supplements like probiotics. The sterile environment of the neonatal intensive care unit (NICU) also alters the microbiome, and neonates in this environment are often there due to pathologies like NEC, sepsis, and infections that are treated with antibiotic medications. Created in BioRender. Sha, C. (2024) BioRender.com/d14m775.

***Breastmilk*** consists of several bioactive components, including immunoglobulins, cytokines, growth factors, hormones, human milk oligosaccharides (HMOs), and microbes that all contribute to the maturation of a neonate’s microbiome [36,37,38]. Although the microbiome of breastmilk is stable and constant across time within an individual, the actual composition and pathophysiology of breastmilk microbiology is a major area of study [37,39]. Hunt et al. report that Firmicutes (*Staphylococcaceae* and *Streptococcaceae*) predominate, while Pannaraj et al. found higher concentrations of Proteobacteria (predominantly *Moraxellaceae* and *Enterobacteriaceae*) [37,40,41,42]. Additionally, formula-fed infants have a distinct gut microbiome profile from breastfed infants [43,44,45]. Backhed et al. found that breastfed infants have a higher abundance of *Bifidobacterium* [29], whereas infants who are formula-fed have more *Clostridium* and *Bacteriodes* in their microbiomes [44]. Nevertheless, this evidence suggests that breastmilk and formula play a formative role in the development and maturation of neonatal microbiomes (Table 2). All the neonatal exposures mentioned in this section are graphically summarized in Figure 1.

**Table 2:** **Factors that affect microbiome composition.** Various early neonatal exposures affect the bacterial composition of the gut microbiome in neonates. These exposures include the conditions of birth (preterm vs. term, C-section vs. vaginal), medications and supplements that affect bacteria (antibiotics, probiotics), nutrition (breastfed vs. formula-fed), and pathology (NEC). The changes indicated are not exhaustive and primarily include the major bacterial phyla and genera affected.

	Preterm [33,46]	C-section [21,23]	Antibiotics [34,35]	Probiotics [47]	Formula [29]	NEC [48]
**Firmicutes**	↓	↑	↑		↑	↓
***Lactobacilli***	↓	↓		↑	↓	
***Staphylococcus Streptococcus Enterococcus***	↑	↑			↑	
***Clostridium***	↓				↑	
**Bacteroidetes *Bacteroides***		↓	↓			
**Proteobacteria**	Depends on genus	Depends on genus	↑		↑	↑
**Actinobacteria**	↓	↓	↓		↓	
***Bifidobacteria***	↓	↓		↑	↓	

## 3. Gut–Microbiota–Brain Axis Signaling

In addition to the ***microbiome***, there are multiple interactions between the gastrointestinal tract and the brain: the ***nervous***, ***endocrine***, and ***immune*** systems. In the following section, the function and early development of these gut–brain pathways will be explored.

### 3.1. Microbiome and Neurodevelopment

Microbiome composition affects systematically-released metabolites that can then affect multiple organ systems, including the brain, heart, liver, and adipose tissue [49]. The importance of these relationships is demonstrated in germ-free animal models, which are bred and maintained in isolated environments. These mice exhibit significant alterations in neurodevelopment, as well as microbiome metabolic divergence in amino acid, carbohydrate, fatty acid, and urea metabolism pathways, when compared to controls [49]. Germ-free mouse pups exhibit lower neuronal network density and connectivity at 4 weeks of age. As they mature, these mice exhibit blunted network refinement and maturation, decreased connections between brain regions, and changes in neurotransmitter expression patterns [50,51]. Behaviorally, germ-free mice display a longer freezing response during contextual fear conditioning, suggesting exaggerated fear memory expression [50]. Microbiota from low-growth versus high-growth preterm infants differentially affected expression of neuronal and myelination markers. Furthermore, Nos1, a synthase implicated in neurotoxicity and neurodegeneration, was increased in mice exposed to low-growth microbiota [52]. Therefore, gut–microbiome–brain interactions are important for normal neurodevelopment.

### 3.2. Nervous System: Autonomic

The human nervous system is divided into the central nervous system (CNS) and the peripheral nervous system (PNS), both of which develop from the neural tube around week 3-4 of gestation [53]. The CNS, comprising the brain and spinal cord, develops from the neural tube. Neural crest cells, derived from the lateral folds during neural tube closure, migrate peripherally and give rise to the PNS, as well as other tissues [54]. Most CNS and PNS development occurs during gestation; however, a significant amount of nervous system growth and maturation, including synaptogenesis, axon pruning, and network refinement, occurs postnatally [55]. It is the PNS which serves as a major conduit of information in the gut–microbiota–brain axis.

The PNS is divided into the autonomic and somatic (or voluntary) nervous systems; however, it is the autonomic nervous system that mediates gut–brain interactions via the enteric, sympathetic, and parasympathetic nervous systems. The enteric nervous system (ENS) is intrinsic to the GI tract and mediates GI motility and function via a network of interneurons, motor neurons, and afferent neurons. Because of its location within the GI wall, the ENS lies near the gut microbiota [56]. Therefore, ENS migration and development into submucosal and myenteric plexuses are vulnerable to any pathological perturbations in the microbiome [57,58]. Perturbations in the ENS function are associated with neurological disorders, such as autism spectrum disorder; however, the specific mechanisms are still unknown and are a major focus of research [59].

ENS activity is modulated by the sympathetic and parasympathetic nervous systems, whose influence is widely distributed: they affect all the organ systems of the body, including the lungs, heart, liver, and gut [60,61]. Spinal sympathetic fibers synapse upon the paravertebral ganglion, which then synapse upon and mediate an inhibitory effect upon the ENS. Parasympathetic input, mediated primarily via the vagus nerve (CN X) and the pelvic plexus, form autonomic plexuses prior to innervating the GI tract. Although the vagus nerve is fully developed and myelinated by birth, the brain regions that regulate the vagus nerve continue to develop postnatally [62]. Myelinated nerves are vulnerable to perinatal disturbances, such as environmental toxins, nutritional deficiencies, or illness, which can predispose individuals to neurological or neuropsychiatric dysfunction later in life [63,64]. Normal vagal development and function, and, presumably, parasympathetic input to the gastrointestinal tract, are associated with healthy infant development and socioemotional behavior [62,65].

### 3.3. Endocrine System

Due to its role in metabolism, the gut microbiome itself can be considered a component of the endocrine system; however, enteroendocrine cells (EECs) lining the intestinal wall also release hormones and metabolites systemically [66]. Although they comprise 1% of intestinal cells, the different EECs release over 30 different gut hormones that regulate other tissues, such as the pancreas, liver, and brain [67,68]. Gut hormones released by the EEC, including ghrelin, glucagon-like peptide 1, insulin-like peptide 5, and peptide Y, affect the CNS by regulating feelings of hunger and satiety [69]. Interestingly, fermentation products released by the gut microbiome regulate and modulate EEC gut hormone production and release [70]. Developmentally, the origin of EECs is unclear; some research suggests a neural crest origin, while other research points to endoderm-derived intestinal crypt pluripotent stem cells [71]. How these cells differentiate into different cell types and guide region-specific hormone expression in the gut appears to be under the control of multiple transcription factor pathways (i.e., Notch, zinc-finger, homeodomain) [68].

### 3.4. Immune System: Inflammatory Mediators

Unlike other organs in the body, the GI tract has direct exposure to the external environment and, therefore, any potential foreign insult. Therefore, the GI system must protect itself and act as a barrier against pathogens, which may include the gut microbiome itself if microbes invade the gut epithelial barrier or release harmful metabolites, such as LPS. Protection often occurs through inflammatory signaling and activation of immune cells. Out of all the cells lining the GI tract, intestinal epithelial cells are the primary cells responsible for releasing inflammatory mediators, such as cytokines and chemokines [72,73]. After release, these signaling molecules, which include tumor necrosis factor-β, interleukin-1β (IL-1β), and IL-6, can activate the innate and adaptive immune responses [74]. Immune cells, including T and B cells, have been shown to infiltrate the brain and interact with brain microglia during pathological disease states [75,76,77]. Prior research demonstrated both neuroprotective and damaging roles of B cells in the brain, depending on the underlying condition [78,79]. Cytokines themselves can travel systemically and traverse the blood brain barrier, where they can interact with neurons and glial cells and affect brain function [80]. Between cytokine release and immune cell activation, disruptions to inflammatory homeostasis in the GI system can result in the neurological sequelae of disorders such as IBD in adults and NEC in infants [81].

The microbiome, autonomic nervous system (enteric, sympathetic, and parasympathetic), endocrine system (enteroendocrine), and immune system (cytokines and cell-mediated) form four presumably bidirectional pillars of normal gut–microbiota–brain axis homeostasis. The next section explores how perturbations within these systems in an immature gut–brain axis can have long-term developmental and functional consequences.

## 4. Gut–Microbiota–Brain Axis Disruption in Neonatal and Pediatric Patients

Among the pediatric population, gut–microbiota–brain axis dysfunction is often found in preterm infants. Preterm infants have immature organ and immune systems, which predispose them to injury and infection. In addition, prolonged stays within the neonatal intensive care unit (NICU) affect gut development because of the abnormal hospital microbiome, increased antibiotic use, and additional nutritional needs, all of which contribute to abnormal GI colonization in a preterm infant.

### 4.1. Vulnerabilities of an Immature Gut

Underdevelopment of the GI and immune systems may predispose preterm infants to NEC [82]. NEC is a devastating disease of prematurity characterized by loss of epithelial integrity, abnormal translocation of gut bacteria, and an inflammatory response by an immature immune system. Although the specific mechanisms are unknown, the convergence of these factors activates innate the immune system’s toll-like receptors, which results in intestinal necrosis, a systemic inflammatory reaction, and sepsis [83]. Preterm infants have decreased gut motility, increased bowel permeability, and reduced protective mucosa, which make them more susceptible to bacterial translocation and NEC [82,84]. Exaggerated inflammatory responses to bacterial activation of toll-like receptors mediated by proinflammatory cytokines like IL-8,have been noted in the gut of premature infants [85]; proinflammatory cytokines produced by epithelial cells also upregulate the production of nitric oxide and reactive oxygen species, which contributes to increased apoptosis and decreased proliferation of epithelial cells [85]. Immature GI tracts are more susceptible to perturbations in the microbiome and have exaggerated and ineffective inflammatory and immune responses.

### 4.2. Abnormal Microbial Colonization of the Gut

The microbiota composition of preterm and low birthweight infants who remain in the NICU diverges significantly from that of full-term infants. This is thought to result from the perinatal hospital environment predisposing preterm infants to abnormal and pathogenic colonization of the microbiome. For example, *Clostridium* colonization, which delays normal anaerobic bacterial colonization, was observed in 79% of NICU preterm infants [86]. Although the clostridial strains were not antibiotic-resistant, this study found that antibiotic courses did not affect the incidence of colonization by *Clostridium,* but rather only decreased the degree of colonization. Preterm infants are observed to have higher abundance of *Enterobacter* [31], along with a decrease in overall microbial diversity [87]. This difference in microbiome composition is further heightened in preterm infants who develop NEC during 2 to 3 weeks prior to disease diagnosis [48]. Analysis of the functional contributions of the bacteria present in the fecal samples found that differences amongst the groups were primarily associated with carbohydrate metabolism. Such findings suggest possible deviances in metabolic pathways due to altered microbiota diversity and composition in NEC patients [48]. *Escherichia*, another family of the Gammaproteobacterium class, was found to be significantly lower in the stool samples of low-birthweight infants when compared to normal-birthweight infants [88]. Preterm birth with perinatal antibiotic use was associated with reduced levels of the genus *Bifidobacterium*, which helps prevent pathogenic colonization of the infant gut [32,89]. While the exact mechanism is not well understood, there are considerable overlaps in the patterns of dysbiosis associated with prematurity and NEC.

Probiotics show potential in mitigating GI pathology in NEC and prematurity. Probiotic supplementation, especially with multi-strain formulations, has been associated with significant reductions in NEC incidence and mortality amongst infants in the NICU when compared with those in the placebo control group [90,91,92]. However, not all studies demonstrate a clear benefit from probiotic supplementation, and the lack of standardization regarding dosing, delivery, and strains administered, along with the risk of probiotic-associated sepsis, prevents probiotics from being a mainstay prophylactic [93,94]. In clinical trials where probiotics were protective against NEC, potential mechanisms include probiotics enforcing epithelial tight junctions, producing biofilms to promote more probiotic attachment, releasing beneficial metabolites, and inhibiting pro-inflammatory pathways [93]. Probiotic administration studies reveal a reduction in the expression of the proinflammatory cytokines IL-1β, IFN-γ, and IL-8, indicating potential inhibition of these proinflammatory cytokines [95].

Beyond NEC, probiotics demonstrate potential therapeutic roles in neurodevelopmental disorders such as autism spectrum disorder (ASD) and attention deficit hyperactivity disorder (ADHD) [96]. One study found that, upon receiving probiotic supplementation for 3 months, the stool of pediatric patients with ASD displayed increased colonization of *Lactobacilli* and *Bifidobacteria*—two species known to lower intestinal pH, regulate the immune response, and reduce pathogenic colonization—with improvements in ASD symptom severity [47]. ‘Normalization’ of the microbiome may help prevent pathogenic colonization of preterm infants and subsequent negative consequences.

### 4.3. Differential Nutritional Needs: Diet and the Microbiome

In addition to the higher nutritional requirements of prematurity, these infants can have other conditions, such as infection, that further increase nutritional needs. Although breastmilk is widely accepted as the recommended form of nutrition because it contains immune-protective factors, growth factors, and favorable protein and lipid profiles, the caloric density can be insufficient for complex preterm infants [97,98,99]. Although attempts to compensate for these nutritional deficiencies have been made with fortifiers, some cow milk-derived fortifiers may actually increase the risk of NEC due to inflammation brought about by the introduction of foreign cow proteins [100]. Similarly, although early enteral feedings are encouraged to optimize nutritional content, these may stress the immature GI system of preterm infants and are a risk factor for abdominal distention and emesis, symptoms that manifest in NEC [101]. In contrast, animal models of NEC find that delayed initiation of enteral feedings reduces NEC incidence [102]. There is wide variability in the practice of initiation and use of enteral feeds in preterm infants, and it is a major area of discussion and research [103].

Nutrition and the gut microbiome have also been implicated in pediatric epilepsy [104]. Human and animal studies have found differences in the gut microbiome of individuals with epilepsy compared to individuals without epilepsy [105,106]. Peng et al. found differences in gut microbiome composition between patients with drug-resistant epilepsy and patients with drug-sensitive epilepsy; specifically, those with drug-resistant epilepsy exhibited greater amounts of Firmicutes bacteria [107]. Recently, ketogenic diets have been shown to be beneficial in attenuating seizures in mice and rats [108,109]. These diets change the gut microbiome and, specifically, increase Verrucomicrobia and decrease *Bacteroides* [108,109]. These findings are further supported with randomized clinical studies that demonstrated a reduction in the number of seizures experienced, by about 40–50%, in children with treatment-resistant epilepsy with a ketogenic diet [110,111].

### 4.4. Disruption Impacts Neurodevelopment

Early brain development is characterized by critical time windows in which vulnerability to disease and alterations from adverse events are especially high. Postnatal infections in preterm infants, in particular, pose a risk factor for later neurodevelopmental disabilities and widespread brain abnormalities. Perinatal brain injury can result in periventricular leukomalacia and cerebral white matter injury that are correlated with neurodevelopmental deficits [112]. NEC and infections, such as sepsis, pneumonia, and meningitis, in preterm infants are associated with significant white matter changes, as well as increased motor, sensory, and cognitive function deficits [113]. Patients who have experienced NEC specifically are at increased risk of neurocognitive impairments, motor deficits, and cerebral palsy [114,115,116]. On cross-sectional imaging, NEC patients demonstrate significantly increased brain injury scores, which includes white matter injury, porencephaly, and ventriculomegaly [117,118].

One major route between the GI tract and the CNS is via intravascular molecules. NEC patients have elevated levels of inflammatory cytokines, including IL-1β, IL-2, IL-6, TNF-α, IFN-γ, and CXCL1 [119,120]. These data have also been replicated in animal studies whereby cytokine levels are elevated in the context of NEC induction. In the brain, these cytokines can directly interact with neurons and glial cells to affect their replication and development. Animal studies have shown that cytokines, such as IFN-γ, affect synaptic function, which can lead to learning deficits [121]. Furthermore, CXCL1 has been implicated in oligodendrocyte maturation [122], which can potentially lead to white matter deficits. It has been shown that NEC-induced inflammation, both in patients and in experimental models, is sufficient to activate microglia [123]. In addition to their role in responding to inflammation and infection, microglia play an integral part in synaptic pruning, cortical refinement, and synaptic plasticity and neurodevelopment [11]. These cytokines and their role in the activation of neuroinflammatory cells and pathways have the potential of disrupting normal neurodevelopment, with subsequent deleterious effects upon cognition [124]. Other facets of the gut–microbiome–brain axis that are affected by NEC are also a major focus of research [83,125].

Finally, apart from neonatal microbiome dysfunction in NEC, the pediatric microbiome dysfunction seen in IBD, Crohn’s disease, and ulcerative colitis is also associated with neuropsychiatric symptoms—anxiety, depression, and memory deficits—both during flare-ups and after the disease is managed [126]. Cognitive functional impairments in NEC and IBD may be due to pro-inflammatory affects upon neurogenesis. A pediatric IBD mouse model study found that acute colitis resulted in behavioral and cognitive deficits, accompanied by neuroinflammation, greater microglia activation, and reduced neurogenesis in the hippocampus, even after overcoming the acute disease [127]. Such findings highlight the long-term consequences that microbiome dysbiosis and activation of the systemic inflammatory cascade can potentially have upon neurodevelopment.

## 5. Conclusions

**Figure 2:**
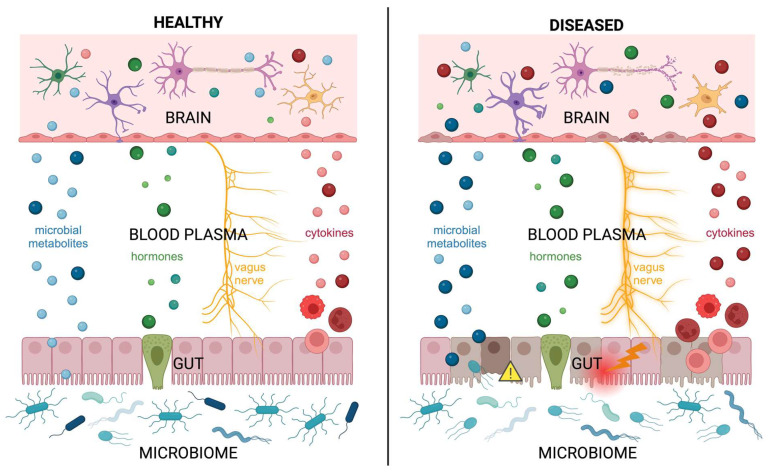
**Signals from the gut traveling along the gut–microbiota–brain axis in healthy versus diseased states.** In healthy states (**left**), the body maintains everything in balance, including the microbiome, metabolites, hormones, cytokines, etc. Following intestinal injury disease (**right**), this balance is disturbed on all levels in the gut–microbiota–brain axis. First, the microbiome changes its composition and has decreased microbial diversity. Changing the bacterial composition of the microbiota then affects the metabolites it releases; gram-negative bacteria tend to release lipopolysaccharide (dark blue, large), which can lead to intestinal damage, immune activation, and sepsis. Beneficial microbial metabolites that promote neurodevelopment, like short-chain fatty acids (light blue, small), are released in lesser quantities. Microbial metabolites also directly influence and perturb balanced hormone (green) release by enteroendocrine cells. Damaged intestinal epithelial cells (light and dark brown) will lose their tight junction integrity and have increased mucosal permeability (yellow caution triangle). The peripheral nervous system, and specifically the vagus nerve, is activated in response to intestinal damage and is a direct signaling pathway to the brain. Gut damage, as well as microbial metabolites like lipopolysaccharide, also activates the immune system, resulting in the release of pro-inflammatory cytokines (dark red, large), disturbing the balance between pro- and anti-inflammatory (light red, small) signals. These mediators travel systemically and can bypass the blood–brain barrier, especially when the brain is immature. During early human development, the brain is particularly vulnerable to these imbalances, resulting in long-term neurodevelopmental and cognitive deficits when these harmful signals interact with cells (oligodendrocyte-green, astrocyte-purple, neuron-pink, microglia-yellow) and the blood–brain barrier. Amongst pediatric diseases, prematurity with nutritional imbalances and necrotizing enterocolitis are poignant examples of how gut injury alters the microbiome and leads to negative systemic imbalances that severely impact neurodevelopment. Created in BioRender. Sha, C. (2024) BioRender.com/r60o312.

In this literature review, we explored the developing gut and its microbiome, as well as how this system signals systemically to affect neurodevelopment and the central nervous system function. Gut development encompasses not only maturation of the gut epithelium but also colonization with the microbiome. The organisms that colonize the microbiome vary depending on early life exposures, which include vaginal or cesarean delivery, antibiotic administration, and the nutrition provided—breastmilk, formula alone, or formula with supplements and probiotics. Different colonization of the microbiome leads to differences in the metabolites produced and released, which can travel throughout the body to the brain and influence early neurodevelopment. Additionally, the gastrointestinal tract communicates and interacts with the brain via the peripheral nervous system (specifically the autonomic nervous system and vagus nerve), endocrine system and enteroendocrine cells, and immune system (both cellularly and chemically). Disruptions to the gut–microbiota–brain axis (Figure 2), especially in early life, are detrimental to proper central nervous system development and maturation and result in dire cognitive and neurological consequences for patients. Therefore, further study in understanding how the gut microbiome develops and the processes underlying the development and refinement of the gut–microbiota–brain axis is critical for treating patients with disruptions to the gut microbiome and preventing subsequent neurodevelopmental consequences.

## Figures and Tables

**Table 1:** Microbiome composition throughout life. Early life (<3 years) microbiome is primarily composed of *Bacteroides* and gram-negative organisms [29]. Beyond the first 3 years of life, Firmicutes become the predominant bacterial species in the gut microbiome of children and adults, followed by Bacteroidetes, Proteobacteria, Actinobacteria, and Verrucomicrobia [28].

Colonization [29]	Infants, 4 Months [29]	Infants, 12 Months [29]	Children, 9 Years [28]	Adults [28]
Bacteroidetes *Bacteroides* (30%)	Actinobacteria *Bifidobacteria* (42%)	Bacteroidetes *Bacteroides* (45%)	Firmicutes (57%)	Firmicutes (75%)
Proteobacteria *Escherichia*/*Shigella* (22%)	Bacteroidetes *Bacteroides* (24%)	Firmicutes *Ruminococcus* (13%)	Bacteroidetes (30%)	Bacteroidetes (13%)
Actinobacteria *Bifidobacteria* (22%)	Proteobacteria *Escherichia*/*Shigella* (5%)	Firmicutes *Clostridium* (5%)	Proteobacteria (6%)	Proteobacteria (5%)
			Actinobacteria (4%)	Actinobacteria (4%)
			Verrucomicrobia (2%)	Verrucomicrobia (1%)

## Data Availability

Data sharing is not applicable.
